# Clinical trials of TB preventive treatment: a scoping review

**DOI:** 10.5588/ijtldopen.26.0255

**Published:** 2026-11-13

**Authors:** T. Hasan, E. Medcalf, A. Kanchar, D. Falzon, B.J. Marais, G.J. Fox

**Affiliations:** 1 The Faculty of Health and Medicine, The University of Sydney, Sydney, NSW, Australia;; 2 Sydney School of Public Health, The University of Sydney, Sydney, NSW, Australia;; 3 Department for HIV, Tuberculosis, Hepatitis and Sexually Transmitted Infections, World Health Organization, Geneva, Switzerland;; 4 Royal Prince Alfred Hospital, Camperdown, NSW, Australia.

**Keywords:** tuberculosis, isoniazid, rifampicin, rifapentine, levofloxacin, randomised controlled trial

## Abstract

**BACKGROUND:**

TB preventive treatment (TPT) is an important strategy to reduce TB incidence in high-risk populations. This scoping review assessed the reporting of published randomised controlled trials (RCTs) evaluating clinical outcomes of regimens to treat TB infection (TBI).

**METHODS:**

We searched PubMed, Cochrane Library, and EMBASE for English-language RCTs published between 2000 and 2025. Eligible studies were Phase 3 or 4 RCTs enrolling individuals receiving TPT for suspected TBI, regardless of drug resistance. Data regarding trial design, population characteristics, efficacy outcomes, and adverse effects were extracted.

**FINDINGS:**

Of 6,327 records screened, 46 trials were identified. Isoniazid monotherapy was most frequently evaluated (n = 14, 30.4%), followed by rifapentine-containing regimens (n = 11, 23.9%). The most common outcome assessed was development of TB disease in the follow-up period (n = 31, 67.4%). In all but four trials, TPT was found to be more efficacious than placebo or no treatment (91.3%). Information about adverse events was collected in 37 studies (80.4%); however, there was significant heterogeneity in the methods by which adverse events were reported.

**CONCLUSION:**

We identified considerable heterogeneity in the regimens investigated as well as the outcomes reported. In general, TPT regimens demonstrated good efficacy, but adverse events and patient-reported outcomes were inconsistently reported.

It is estimated that nearly a quarter of the global population is infected with *Mycobacterium tuberculosis*, the cause of TB.^1^ Around 5%–10% of people with TB infection (TBI) will develop TB disease during their lives, with those recently infected or re-infected being at greatest risk.^2^ The End TB Strategy aims to reduce global TB incidence by 90% by 2035,^3^ which will require earlier treatment of people with TB disease and reducing the large reservoir of TBI from which new cases arise.^4^ TB preventive treatment (TPT) reduces the risk of TB disease development, but reported efficacy has been highly variable.^5^ The protective effect is highest in low-TB-incidence settings where community transmission and the risk of re-infection is low. TPT has been used in low-prevalence settings for over 40 years. Early trials demonstrated the effectiveness of regimens of between 6 and 18 months of daily isoniazid^6^; however, significant hepatotoxicity and the long treatment duration impeded take up – particularly for older people.

In the last decade, several systematic reviews and meta-analysis have reported TPT to be effective in reducing TB incidence,^7,8^ including in immunosuppressed populations.^9^ However, availability in resource-limited setting remains limited, ‘real life’ adherence is sub-optimal, and adverse events (AEs) are not negligible. In most studies, nearly 5% of individuals cease treatment due to intolerable AEs.^10^ Current WHO recommendations for TPT regimens considered outcomes from various clinical trials and observational studies.^11^ However, to inform optimal global TPT recommendations, data are required from as many different studies and population groups as possible. In 2025, WHO developed Guidance on Evidence Generation (GEG), to inform researchers, developers, funders, and other stakeholders about how evidence should be generated to optimally inform WHO guideline development on novel regimens for TPT.^12^ As a part of this process, WHO commissioned a scoping review of contemporary Phase 3 and 4 TPT trials with a focus on reported outcomes.

We present the findings of this review, with a focus on key knowledge gaps that require consideration when trialists develop the next generation of policy-focused TPT research.

## METHODS

A scoping review, performed in accordance with PRISMA-ScR guidelines for scoping reviews with special attention to preferred reporting items.^13^

### Search strategy and selection criteria

Electronic searches were conducted in PubMed, Cochrane Library, and EMBASE databases for English-language articles published between 1 January 2000 and 31 March 2025. The search was used to identify randomised controlled trials and clinical trials investigating TPT regimens in humans (Supplementary Data 1). The search strategy included Medical Subject Headings (MeSH) terms for ‘tuberculosis’, ‘preventive therapy’, and ‘clinical trial’. Reference lists of identified systematic reviews and meta-analyses were used to identify additional studies. Clinical trials registries, including https://clinicaltrials.gov/, https://trialsearch.who.int/ and https://www.anzctr.org.au/, were searched to identify studies. We included randomised controlled trials (Phase 3 or 4) which enrolled individuals taking TPT, regardless of known or unknown drug resistance profiles. English publications reporting primary and secondary outcomes as well as protocol papers were included. Studies evaluating the effectiveness of treatment for TB disease and TB vaccines were excluded. Full text reviews and data extraction were completed by one reviewer (TH), with all extractions checked by a second reviewer (EM). Discrepancies were resolved in consultation with a third reviewer (GJF).

### Data extraction and analysis

Data extracted for each trial included study design characteristics (including superiority or non-inferiority design and clustering), blinding, the study population (e.g., age group, pregnant women, and people living with HIV), intervention and comparator regimens, study outcomes (including patient-reported outcomes), the target sample size, the follow-up duration, and the cumulative incidence of TB in each group. Results were presented using descriptive statistics. Meta-analyses of included studies were not performed, on account of the heterogeneity of interventions and comparator regimens.

### Ethical statement

This was a scoping review and no ethical approval was required.

## RESULTS

The search strategy identified 6,327 titles and abstracts. After deduplication and screening of 5,033 records, 197 full-text articles were reviewed, and 46 eligible trials were included in the systematic review. The PRISMA flow chart is presented in Figure. Published clinical trials demonstrated notable heterogeneity in primary outcome measures reported, as well as definitions of secondary outcomes, which complicated comparison.

**Figure.**
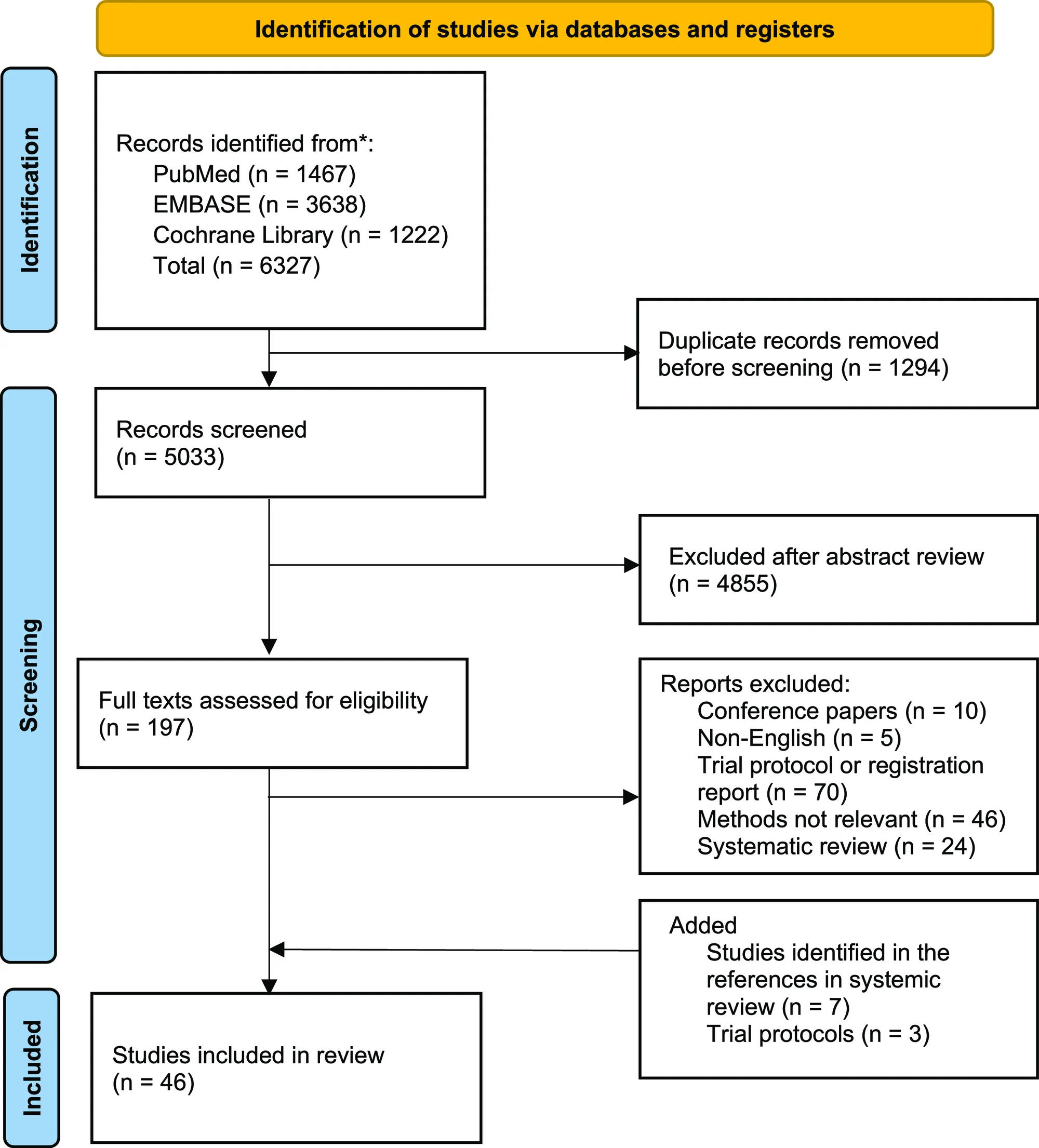
PRISMA flow chart for the identification of randomised controlled trials investigating TB preventive treatment.

### TPT trial characteristics

Of the 46 trials, 14 (30.4%) each were conducted in Africa^14–27^ or Asia,^28–41^ 5 (10.9%) each in Europe^42–46^ or the Americas,^47–51^ and 1 in Australia.^52^ Only seven (17.4%) were multi-centre trials conducted in multiple world regions^53–59^ (Supplementary Data Table S1). People living with HIV were enrolled in 24 (52.2%) studies,^14–23,25–27,33,40,42,47,51,53–57,59^ with 16 (34.8%)^14–17,19–23,25,27,33,42,51,55,56^ exclusively focused on this group. Individuals under 18 years were included in 12 (26.1%) studies,^16,18,21,24,26,27,39,40,43,44,58,59^ and 7 (15.2%)^16,18,21,24,26,44,58^ focused solely on children (<15 years). Only one study (2.2%) included pregnant women,^56^ or focused on older individuals (50–69 years).^35^ Some studies included people with co-morbidities such as diabetes (eight studies, 17.4%),^32,34,35,38,40,41,46,50^ hepatitis C virus infection (seven studies, 15.2%),^30–32,40,41,49,53^ and renal transplantation (two studies, 4.3%).^29,31^ The diagnosis of TBI varied widely across studies (Supplementary Data Table S1). In 16 (34.8%) studies, individuals were included in the setting of immunosuppression (e.g., people living with HIV or transplant recipients), without any other criteria, once active TB was excluded.^15,16,18,20–25,27,29–31,33,55,56^ TBI was diagnosed by tuberculin skin test (TST) in 13 (28.2%) studies,^14,19,28,32,36,40,43,44,48,50,51,53,59^ by interferon-gamma release assay (IGRA) in 3 (6.5%) studies,^34,35,38^ by IGRA or TST in 1 (2.1%) study,^46^ based on contact history alone in 3 (6.5%) studies,^26,39,41^ or by local, national, or other guidelines in 7 (15.2%) studies.^37,45,48,49,52,57,58^

### TPT regimens used

Supplementary Data Table S1 provides an overview of the regimens used in different studies. Isoniazid monotherapy was used in 14 (30.4%) trials,^16–18,20–22,24,30,31,33,34,43,47,56^ of which two enrolled only people living with HIV, comparing 36 months of isoniazid with 6 months of daily isoniazid.^20,33^ A single study compared daily isoniazid use during pregnancy against isoniazid given post-partum for TBI.^56^ The remaining 11 studies investigated isoniazid daily for 6–12 months’ (mostly 6 months’) duration against placebo or no treatment.^16–18,21,22,24,30,31,34,43,47^ Two (4.3%) studies among people with HIV assessed the effectiveness of anti-retroviral therapy with or without TPT (isoniazid or isoniazid and rifapentine) used in combination.^23,25^ Other studies evaluated rifampicin alone^29,32,49,50,54,57,58^ (seven studies, 15.2%), rifampicin and pyrazinamide^28,48,51^ (three studies, 6.5%), isoniazid and rifampicin^45^ (one study, 2.2%), and various combinations of weekly or twice weekly isoniazid with rifapentine for 1–3 months (11 studies, 23.9%).^27,36–39,41,46,52,53,55,59^ Six (13.0%) studies^14,15,19,35,42,44^ investigated multiple TPT regimens. Isoniazid for 6–12 months was the most common comparator (21 studies, 45.7%),^14,19,27–29,32,36,39,44,45,48–55,57–59^ followed by no regimen (4 studies, 8.7%),^35,37,38,42^ placebo (1 study, 2.2%),^15^ 3 months of isoniazid and rifapentine (1 study, 2.2%),^41^ and rifampicin and isoniazid (1 study, 2.2%).^46^ In addition, two (4.3%) studies^26,40^ investigated 6 months of levofloxacin as TPT against placebo in contacts of patients (adults and children) with multidrug- or rifampicin-resistant TB. These studies uniquely also performed a combined individual patient-data meta-analysis and weighted Bayesian analysis, reported in a complementary publication.^60^

### Reporting of TPT trial outcomes

The primary and secondary outcomes of each study are presented in Supplementary Data Table S2. The most frequently assessed primary outcome was the incidence of microbiologically or clinically confirmed incident TB, reported in 26 (56.5%) studies.^14–22,26,29–31,33–35,37,38,40,42,47,51,53,55,57,58^ Overall, 31 (67.4%) studies^14–22,26,27,29–31,33–35,37,38,40,42,44,45,47,51,53,55–59^ reported TB incidence as a primary or secondary outcome. The incidence of AEs was assessed as the primary outcome in nine (19.6%) studies^28,32,43,45,49,50,54,56,59^ and either as a primary or secondary outcome in 37 (80.4%) studies.^16–19,21–24,26–30,32,33,35–38,40–46,48–51,53–59^

### Efficacy

Supplementary Data Table S3 shows the reported effect of TPT regimens in relation to incident TB. Of the 31 studies that measured the incidence of active TB, most studies did not specify how many were diagnosed microbiologically versus clinically. In all but four of these (87.1%) studies,^17,27,33,38^ there was a reduction in incident TB with the intervention regimen as compared to the comparator. The degree of protection was variable and reflected in different ways, but TPT was generally associated with a 40%–60% reduction in the risk of incident TB. Eight trials were non-inferiority trials, four investigated 3HP^46,53,55,59^ (three against H and one against 3RH), two investigated 4R^57,58^ (against 9H), one investigated 6H in pregnancy,^56^ and the final trial investigated levofloxacin against placebo.^40^ All these trials met the pre-specified non-inferiority margin.

### Adverse effects

A standardised method for AE reporting, such as Common Terminology Criteria for Adverse Events (CTCAE)^61^ or DAIDS^62^ tables, was used in 24 (52.2%) studies.^16,18,19,21–24,26,27,32,35–38,40,46,50,51,53–58^ Where a validated tool was not used, laboratory cut-offs to define an adverse event were usually specified by the study. Pre-specified AEs of special interest (AESI) were assessed in 33 (71.7%) studies,^17,18,22–24,26–30,32,33,35–38,40–43,45,46,48–51,53–59^ with hepatotoxicity being the most common AESI investigated (30 studies, 65.2%).^17,18,22–24,27–30,32,35–37,40–43,45,46,48–51,53–59^

### Adherence

Treatment completion was the primary outcome for seven (15.2%) studies^27,36,39,44,46,48,49^ and was reported as a secondary outcome in 16 (34.7%) studies.^32,35,37,38,40–43,50,52–55,57–59^ However, methods for assessing treatment completion varied considerably, with some consistency of defining completion by the completion of ≥80% of doses in ≤120% of the allocated time. Treatment adherence was reported in 26 (17.4%) studies,^17,19,21,25–28,32,33,35–39,41,43–46,48–50,53,54,56,59^ with the most common methods of assessing adherence being pill count and directly observed therapy. Only two (4.3%) studies^48,52^ reported costs associated with TPT interventions, and specific patient-reported outcomes were not reported by any included studies.

## DISCUSSION

This scoping review identified 46 randomised controlled TPT trials conducted since the year 2000, investigating a range of regimens. Most trials investigated the effectiveness of the tested TPT regimen against subsequent TB development and measured AEs as secondary outcomes. TPT regimens were generally effective in reducing incident TB, compared to placebo or no treatment, but adverse event and adherence reporting were too variable to make generalisable deductions.

Several trials compared newer shorter TPT regimens (e.g., 3HP, 1HP, and 4R) to isoniazid monotherapy (6H or 9H), but we found few comparisons between novel regimens such as rifapentine-based regimens, which limits direct evidence for the relative efficacy, safety, and tolerance of newer regimens.

While most studies evaluated the effect of TPT regimens upon subsequent incident TB, the criteria for determining TBI (based on tests like TST and QuantiFERON Gold or based on contact history) and the duration of follow-up for incident disease development varied greatly between trials. This inconsistency in the reporting of baseline TBI status and follow-up periods can influence comparisons for subsequent TB incidence rates.

Trials used different approaches to exclude TB. Symptom screening alone has low sensitivity for detecting TB in adults, but is recommended by the WHO in vulnerable children where access to TPT is more important than the identification of ‘asymptomatic disease’.^1,63^ In adult populations, it is estimated that asymptomatic TB may be responsible for up to 50% of incident TB^64^ and contribute up to 68% of global TB transmission.^65^ Methods to exclude prevalent TB before TPT randomisation also showed variability, with several studies reporting minimal details of the specific screening approaches used (e.g., microbiological testing, molecular tests for TB, or chest radiology). These are important details to consider, given increasing awareness of asymptomatic TB as a contributor to the global TB burden^66^ and the risk of TPT drug resistance acquisition if underlying disease is missed, especially in people with multi-bacillary disease.

The follow-up period for most trials was 12–24 months after randomisation. Few reported on long-term progression to active TB, post-treatment morbidity, or mortality. When measuring TPT regimen durability over a longer time period, it is important to consider the relative risk of exogenous re-infection as the cause of disease. This risk is highly setting dependant, and incident disease resulting from exogenous re-infection is more reflective of local TB control than TPT efficacy.^67,68^ In a randomised trial, re-infection should affect both groups equally, potentially biasing findings towards the null. Trials with longer periods of follow-up will be more affected, particularly in high-transmission settings, and it would be interesting to compare efficacy outcomes at different periods of follow-up. In the case of progression to active TB disease, which is microbiologically confirmed, where available assessment of disease using whole genome sequence comparisons may also be of interest to help quantitate the likelihood of re-infection.

Adherence may influence treatment effectiveness, but its measurement differed significantly across trials. Given the importance of adherence for ‘real life’ impact, the limited description of patient information and adherence tools used in clinical trials is a major oversight and may partially explain the limited generalisability of findings. Similar to adherence, harmonised definitions and reporting of treatment completion is important for direct comparability across studies, enabling valid meta-analyses and informing realistic implementation targets. We suggest based on the most commonly reported measures of completion that reporting how many participants completed ≥80% of required doses within ≤150% of the specified treatment time would facilitate comparison of successful treatment completion.

While many included studies reported on AEs and severe adverse events, the consistency and detail of reporting varied. As individuals with TBI are generally healthy, even low-grade AEs may influence decisions to discontinue TPT. Willingness to accept a small risk of higher-grade AEs may also be greatly reduced, which is why comprehensive AEs are such a critical outcome measure. Surprisingly few non-hepatotoxicity-related AESIs were reported, of which hypersensitivity reactions with rifamycin use is an example.^53,55^

Most studies excluded important subpopulations, such as pregnant women, children, and immunosuppressed individuals. Data on pregnant women were particularly limited with a single study specifically focusing on this group.^56^ Expanding eligibility to other under-represented subgroups, such as those at the ends of the age spectrum (e.g., young children and the elderly), those with co-infections beyond HIV (like Hepatitis B or C), or other immunocompromising states (like diabetes or immunosuppression), also requires consideration.

Despite its systematic approach and comprehensive nature, the scoping review was limited by data availability and variability, which limited comparability between studies and precluded informative meta-analyses. Another limitation of the study was the inclusion of English-language studies only, which may exclude some of the data that is available from high-TB settings including China. Despite limitations, most studies included useful clinical, microbiological, safety, and adherence data.

This study has several important implications for researchers designing future trials. Current studies provide clinicians with a wide range of options for treating TPT, but there is an urgent need for trials comparing alternative regimens with one another. Future studies should ideally also include a broader range of outcomes such as pharmacokinetics/pharmacodynamics data, microbiome impacts, patient-reported outcomes, and resources required (health system and patient costs).^69^ In-trial cost effectiveness analyses are particularly important to improve the translation of clinical findings into national TB programmes. Future trials should also be designed to expand eligibility criteria to diverse populations where possible (children, pregnant women, older adults, co-morbidities) to improve generalisability into real life settings.

## CONCLUSION

The use of harmonised approaches to measure key outcomes, such as incident TB and AEs, is crucial with the adoption of internationally standardised tools (such as CTCAE or DAIDS) where they exist. Since large multi-centre trials aim to change global policy and practice, it would be ideal to involve the WHO guideline process early in the design process to align expectations and endpoints, and to ensure optimal sub-study planning and value extraction. Standardised approaches to measuring efficacy outcomes, as well as adherence and AEs, in TPT trials will assist optimal data use, by facilitating informative meta-analyses and collecting the data necessary to change global guidance.

## Supplementary Material
