# Supplementary material for: Clinical trials of TB preventive treatment: a scoping review
Source: IJTLD Open. 2026 Nov 13;3(11):701–7. doi: 10.5588/ijtldopen.26.0255 (PMC13763976; doi:10.5588/ijtldopen.26.0255)
Supplement: Supplementary file 1 [file ijtldopen26-0255_supplementarydata1.pdf]

Supplementary appendix 1

Search terms PubMed

1. Latent infection
2. Latent Tuberculosis [MeSH]
3. Latent tuberculosis
4. TB infection
5. Latent TB
6. Latent TB infection
7. LTBI
8. Mycobacterium tuberculosis

AND

8. TPT
9. Preventative therapy
10. Prophylaxis
11. Treatment
12. Isoniazid [MeSH]
13. Isoniazid
14. INH
15. 6H
16. 9H
17. 6INH
18. 9INH
19. Rifampicin
20. Rifampin [MeSH]
21. Rifampin
22. RIF
23. 4R
24. 4RIF
25. Rifabutin
26. Rifapentine
27. Rifamycin
28. 3HR
29. 4HR
30. 3HP
31. Levofloxacin [MeSH]
32. Levofloxacin
33. Lfx
34. 6Lfx
35. Delamanid
36. Dlm
37. Antituberculosis agent
38. Chemoprevention
39. IPT

AND

39. Clinical trial
40. Randomised control trial
41. Controlled clinical trial
42. Placebo
43. Control
44. Trial

Search terms Cochrane Database of Systematic Reviews

1. Latent infection
2. MeSH descriptor: [Latent tuberculosis] explode all trees

3. Latent tuberculosis
4. TB infection
5. Latent TB
6. Latent TB infection
7. LTBI
8. Mycobacterium tuberculosis

AND

8. TPT
9. Preventative therapy
10. Prophylaxis
11. Treatment
12. MeSH descriptor: [Isoniazid] explode all trees
13. Isoniazid
14. INH
15. 6H
16. 9H
17. 6INH
18. 9INH
19. Rifampicin
20. MeSH descriptor: [Rifampin] explode all trees
21. Rifampin
22. RIF
23. 4R
24. 4RIF
25. Rifabutin
26. Rifapentine
27. MeSH descriptor: [Rifamycins] explode all trees
28. Rifamycins
29. 3HR
30. 4HR
31. 3HP
32. MeSH descriptor: [Levofloxacin] explode all trees
33. Levofloxacin
34. Lfx
35. 6Lfx
36. Delamanid
37. Dlm
38. MeSH descriptor: [Antitubercular Agents] explode all trees
39. MeSH descriptor: [Chemoprevention] explode all trees
40. IPT

AND

40. Clinical trial
41. Randomised control trial
42. Controlled clinical trial
43. Placebo
44. Control
45. Trial

Search terms Embase

1. Latent infection
2. exp Latent Tuberculosis/
3. Latent tuberculosis
4. TB infection
5. Latent TB
6. Latent TB infection

7. LTBI
8. Mycobacterium tuberculosis

AND

8. TPT
9. Preventative therapy
10. Prophylaxis
11. Treatment
12. exp Isoniazid
13. Isoniazid
14. INH
15. 6H
16. 9H
17. 6INH
18. 9INH
19. Rifampicin
20. exp Rifampin
21. Rifampin
22. RIF
23. 4R
24. 4RIF
25. Rifabutin
26. Rifapentine
27. Rifamycin
28. 3HR
29. 4HR
30. 3HP
31. Exp levofloxacin
32. Levofloxacin
33. Lfx
34. 6Lfx
35. Delamanid
36. Dlm
37. Antituberculosis agent
38. Chemoprevention
39. IPT

AND

39. Clinical trial
40. Randomised control trial
41. Controlled clinical trial
42. Placebo
43. Control
44. Trial

Table S1: Description of tuberculosis preventive treatment (TPT) trials (January 2000 - March 2025)

Author	Setting(s)	Blinding	Study design [Phase]	Regimens (intervention vs control)	Inclusion criteria	Age range (years)	%children (Under 15, unless otherwise stated)	%people living with HIV	Other vulnerable populations included	Sample size* (n)	Female sex (%)
**Isoniazid-monotherapy**											
**Fitzgerald et al 2000** [1]	Haiti	Nil	RCT [phase 3]	12H vs placebo	≥18 years, post primary TB disease	≥18	0	61	NA	233	49·8
**Agarwal et al 2004** [2]	India	Nil	Randomised trial [phase 3]	12H vs no treatment	People with ESRF renal transplant	15 to 58	0	0	Renal transplant	85	7·4
**Vikrant et al 2005** [3]	India	nil	RCT [phase 3]	12H vs no treatment	≥14 years on renal replacement therapy	16-53 for 12H; 14-62 for control arm	0	0	ESRF, HBV, HCV	109	15·6
**Naqvi et al 2006** [4]	Pakistan	nil	Randomised trial [phase 3]	9H vs no treatment	Renal allograft recipients	NA	0	0	Renal transplant, HCV	480	NA
**Zar et al 2007** [5]	South Africa	Participant, clinician, analyst	RCT [phase 3]	H vs placebo	≥8 weeks old neonates with HIV who are contacts of TB cases	≥8 weeks	100[+]	100	NA	263	44
**Mohammed et al 2007** [6]	South Africa	Participant, clinician, analyst	RCT [phase 3]	12H vs placebo	PLHW with negative TST	≥18	0	100	NA	118	50·8
**Madhi et al 2011** [7]	South Africa, Botswana	Participant, clinician, analyst	RCT [phase 2/3]	H vs placebo	HIV exposed infants 91 to 120 days	91 to 120 days	100[+]	40·4	NA	1352	52

Author	Setting(s)	Blinding	Study design [Phase]	Regimens (intervention vs control)	Inclusion criteria	Age range (years)	%children (Under 15, unless otherwise stated)	%people living with HIV	Other vulnerable populations included	Sample size* (n)	Female sex (%)
**Samandari et al 2011** [8]	Botswana	Participant, clinician, analyst	RCT [phase 3]	36H vs 6H + placebo	≥18 years PLWH	IQR 28-39^	0	100	NA	1995	72
**Swaminathan et al 2012** [9]	India	Analyst	RCT [phase 3]	6H vs 36H	≥18 years PLWH	≥18	0	100	NA	712	63
**Gray et al 2014** [10]	South Africa	Participant, clinician, analyst	RCT [phase 3]	H vs placebo	≥8 weeks infants with HIV	IQR 17-63 months^	100[+]	100	NA	167	50·3
**Kim et al 2015** [11]	South Korea	Analyst	Randomised trial [phase 3]	9H vs no treatment	IGRA positive renal pancreas transplant recipients	≥18	0	0	Diabetes, hypertension	263	35
**Rangaka et al 2015** [12]	South Africa	Participant, clinician, analyst	RCT [phase 3]	12H vs placebo	≥18 years PLWH on ART	IQR 30-40^	0	100	NA	1329	75·1
**Danel et al 2015** [13]	Ivory Coast	nil	RCT [phase 3]	6H + ART vs ART	≥18 years PLWH with CD4 <800 cells/mm^3	IQR 29-42^	0	100	NA	2056	79·5
**Gupta et al 2019** [14]	Botswana, Haiti, India, South Africa, Tanzania, Uganda, Zimbabwe, Thailand	Participant, clinician, analyst	Non-inferiority RCT [phase 3]	6H during pregnancy vs 6H post-partum	≥18 years pregnant women living with HIV	IQR 24-33^	0	100	NA	956	100

Author	Setting(s)	Blinding	Study design [Phase]	Regimens (intervention vs control)	Inclusion criteria	Age range (years)	%children (Under 15, unless otherwise stated)	%people living with HIV	Other vulnerable populations included	Sample size* (n)	Female sex (%)
**LaCourse et al 2021** [15]	Kenya	nil	Randomised trial [phase 4]	12H vs no treatment	HIV exposed infants	6-10 weeks	100⁺	0	NA	298	47·3
**Rifampicin-based regimens**											
**Gordin et al 2000** [16]	US, Mexico, Haiti, Brazil	Nil	RCT [phase 3]	2RZ vs 12H	≥13 years PLWH, with positive TST	16-70	0	100	NA	1583	28·5
**Leung et al 2003** [17]	Hong Kong	Nil	RCT [phase 4]	2RZ vs 6H	People with silicosis and positive TST	mean age 61·6 (in 2RZ arm)	0	0	Silicosis	76	1·3
**Menzies et al 2004** [18]	Canada	Nil	RCT [phase 4]	4R vs 9H	Adults with positive TST	≥18	0	0	NA	116	44
**Tortajada et al 2005** [19]	Spain	Nil	RCT [phase 4]	2RZ vs 6H	Contacts of confirmed TB cases with positive TST	≥1	17·3 (≤19 years)⁺	0	NA	352	49·1
**Menzies et al 2008** [20]	Saudi Arabia, Brazil, Canada	nil	Randomised trial [phase 4]	4R vs 9H	≥18 years with TBI	≥18	0	1·5	NA	847	47·3
**Chan et al 2012** [21]	Taiwan	nil	RCT [phase 3]	4R vs 6H	HIV negative prison inmates with positive TST	≥18	0	0	HCV, diabetes	373	0
**White et al 2012** [22]	United States	nil	RCT [phase 4]	4R vs 9H	Prison inmates with TBI	NA	0	0	HCV	364	7
**Jimenez-Fuentes et al 2013** [23]	Spain	nil	RCT [phase 3]	3HR vs 6H	Immigrants with TBI	12-40	0	0	NA	590	32·2
**Diallo et al 2018** [24]	Australia, Benin, Brazil,	Analyst	Non-inferiority	4R vs 9H	Children with TBI	0-17	100 (≤ 17 years)⁺	0	NA	844	50·3

Author	Setting(s)	Blinding	Study design [Phase]	Regimens (intervention vs control)	Inclusion criteria	Age range (years)	%children (Under 15, unless otherwise stated)	%people living with HIV	Other vulnerable populations included	Sample size* (n)	Female sex (%)
	Canada, Ghana, Guinea, Indonesia, Saudi Arabia, South Korea		RCT [phase 3]								
**Menzies et al 2018** [25]	Australia, Benin, Brazil, Canada, Ghana, Guinea, Indonesia, Saudi Arabia, South Korea	Analyst	Non-inferiority RCT [phase 3]	4R vs 9H	Adults with TBI	18-90	0	4	Immunosuppression	6012	59·1
**Tamez Torres et al 2023** [26]	Mexico	Analyst	RCT [phase 4]	3R vs 6H	≥18 years with diabetes and positive TST	IQR 50-62^	0	0	Diabetes, alcohol, smoker, renal disease	131	58·8
**Rifapentine-based regimens**											
**Sterling et al 2011** [27]	United States, Canada, Brazil, Spain	Analyst	Non-inferiority RCT [phase 3]	3HP vs 9H	≥2 years, close contacts of TB cases with positive TST	IQR 25-46^	0	2·6	HBV, HCV	7731	45·5
**Villarino et al 2015** [28]	United States, Canada, Brazil,	Analyst	Non-inferiority RCT [phase 3]	3HP vs 9H	2-17 years with TBI with positive TST	2-17	25 (≤12 years)⁺	0·5	NA	905	49·1

Author	Setting(s)	Blinding	Study design [Phase]	Regimens (intervention vs control)	Inclusion criteria	Age range (years)	%children (Under 15, unless otherwise stated)	%people living with HIV	Other vulnerable populations included	Sample size* (n)	Female sex (%)
	Spain, Hong Kong										
**Denholm et al 2016** [29]	Australia	nil	RCT [phase 4]	3HP vs 9H	≥18 years with TBI	18-77	0	0	NA	80	51·2
**Gao et al 2018** [30]	China	Analyst	RCT [phase 3]	3HP, $2H_2P_2$ vs no treatment	IGRA positive rural residents aged 50-69 years	50-69	0	0	Diabetes	3738	45·1
**Sun et al 2018** [31]	Taiwan	nil	RCT [phase 3]	3HP vs 9H	≥12 years, close contacts of TB cases with positive TST	≥12	0	0	NA	263	42·2
**Swindells et al 2019** [32]	Ten countries	Analyst	Non-inferiority RCT [phase 3]	1HP vs 9H	≥1 years living with HIV	IQR 28-43^	0	100	NA	3000	54
**Churchyard et al 2021** [33]	South Africa, Ethiopia, Mozambique	nil	RCT [phase 3]	3HP vs 6H	PLWH on ART	IQR 35-49^	0·3 (≤ 18 years)	100	NA	4014	69·6(in 3HP)
**Ruan et al 2021** [34]	China	Analyst	Randomised trial [phase 3]	3HP vs no treatment	≥18 years with silicosis and HIV negative	18-65	0	0	NA	513	0
**Surey et al 2021** [35]	United Kingdom	nil	Non-inferiority RCT [phase 4]	3HP vs 3RH	16-65 years with positive TST or IGRA	16-65	0	0	Diabetes, immunosuppression, alcohol, smoker	52	50
**Chaisson et al 2023** [36]	Uganda	nil	Randomised trial [phase 3]	3HP + ART vs ART	ART naïve PLWH with CD4 <350 cells/mm^3	IQR 25-37^	0	100	NA	453	63·4

Author	Setting(s)	Blinding	Study design [Phase]	Regimens (intervention vs control)	Inclusion criteria	Age range (years)	%children (Under 15, unless otherwise stated)	%people living with HIV	Other vulnerable populations included	Sample size* (n)	Female sex (%)
**Zhang et al 2023** [37]	China	Analyst	RCT [phase 3]	6 weeks H_2P_2 vs nil treatment	18-75 years with TBI positive IGRA	18-75	0	0	HBV, diabetes, alcohol, smoker	677	41·9
**Chen et al 2024** [38]	China	nil	Cluster-RCT [phase 4]	$3H_2P_2$ vs 6H	Close contacts of confirmed TB cases	5-64	12·5	0	NA	2434	58·8
**Huang et al 2024** [39]	Taiwan	nil	Randomised trial [phase 3]	1HP vs 3HP	≥12 years, close contacts of TB cases	≥12	0	0	Diabetes, HBV, HCV	490	50·8
**Trials investigating multiple regimens**											
**Johnson et al 2001** [40]	Uganda	Analyst	RCT [phase 3]	3HR, 3HRZ, 6H vs placebo	PLWH with positive TST	18-50	0	100	NA	2736	NA
**Quigley et al 2001** [41]	Zambia	Nil	RCT [phase 3]	6H, 3RZ vs placebo	≥15 years PLWH	≥15	0	100	NA	1053	NA
**Rivero et al 2003** [42]	Spain	Nil	Randomised trial [phase 3]	6H, 3RH, 2RZ vs nil	PLWH with negative TST	18-65	0	100	NA	319	28
**Spyridis et al 2007** [43]	Greece	nil	Randomised trial [phase 3]	3HR, 4HR vs 9H	≤15 years with contact history and positive TST	0-15	100⁺	0	NA	926	48·6
**Martinson et al 2011** [44]	South Africa	nil	RCT [phase 3]	3HP, 3HR, 6 years H vs 6H	PLWH with positive TST	IQR 26·4-34·7^	0	100	NA	1148	83·3
**Levofloxacin based regimens for MDR/RR-TB**											
**Fox et al 2024** [45]	Vietnam	Participant, clinician, analyst	Non-inferiority RCT [phase 3]	6Lfx vs placebo	Household contacts of MDR/RR-TB cases, of any age with positive TST	IQR 28-52^	2·90	0·4	Diabetes, renal disease, HBV, HCV, HIV, lung disease	2041	64

Author	Setting(s)	Blinding	Study design [Phase]	Regimens (intervention vs control)	Inclusion criteria	Age range (years)	%children (Under 15, unless otherwise stated)	%people living with HIV	Other vulnerable populations included	Sample size* (n)	Female sex (%)
**Hesseling et al 2024** [46]	South Africa	Participant, clinician, analyst	Cluster-RCT [phase 3]	6Lfx vs placebo	Children who are contacts of MDR/RR-TB cases	IQR 1·3 - 4·2^	100⁺	2·1	NA	922	50·8

ART antiretroviral therapy, ESRF end stage renal failure, H isoniazid, H_2P_2 twice weekly isonizid and rifapentine, HBV hepatitis B virus, HCV hepatitis C virus, HIV human immunodeficiency virus, IGRA interferon gamma release assay, IQR interquartile range, MDR/RR-TB multi-drug or rifampicin resistant tuberculosis, P rifapentine, PLWH people living with HIV, R rifampicin, TB tuberculosis, Lfx levofloxacin, TBI tuberculosis infection, TPT tuberculosis preventive therapy, TST tuberculin skin test, Z pyrazinamide

*Combined population of intervention and control arms

⁺Included children under 5 years of age

Table S2: Non-efficacy outcomes reported by Phase 3 and 4 TB preventive treatment TPT trials (January 2000 - March 2025)

Author	Primary outcome	Secondary outcomes	AE reporting classification	Grade 1-5 AE (Y/N)	Grade >3 SAE	Key adverse events	Adherence reporting method	Treatment completion measurement
**Isoniazid monotherapy**								
**Fitzgerald et al 2000** [1]	TB incidence	NA	NA	N	N	NA	NA	NA
**Agarwal et al 2004** [2]	TB incidence	Hepatotoxicity grade ≥ 3	NA	N	N	Hepatotoxicity	NA	NA
**Vikrant et al 2005** [3]	TB incidence	Hepatotoxicity grade ≥ 3	NA	N	N	Hepatoxicity	NA	NA
**Naqvi et al 2006** [4]	TB incidence	NA	NA	N	N	NA	NA	NA
**Zar et al 2007** [5]	TB incidence	Grade 3/4 AE, mortality	DAIDS [47]	Y	Y	NA	NA	NA
**Mohammed et al 2007** [6]	TB incidence	AE of any grade, adherence, mortality, change in CD4	NA	N	N	Hepatotoxicity	Pill count	NA
**Madhi et al 2011** [7]	TB incidence	Mortality, AESI	DAIDS [47]	N	Y	Peripheral neuropathy, hepatotoxicity	NA	NA
**Samandari et al 2011** [8]	TB incidence	Mortality	NA	N	N	NA	NA	NA
**Swaminathan et al 2012** [9]	TB incidence	AE of any grade, mortality	NA	Y	Y	Arthralgia, nausea, vomiting, jaundice, neuropathy, cutaneous reactions	Home visits, pill counts and spot urine samples	NA
**Gray et al 2014** [10]	TB disease, mortality	AE of any grade, hospital admissions, adherence	DAIDS [47] (for paediatrics)	N	N	NA	Pill count	NA
**Kim et al 2015** [11]	TB incidence	Mortality, transplant rejection	NA	N	N	NA	NA	NA
**Rangaka et al 2015** [12]	TB incidence	AE of any grade, mortality, loss to follow up	ACTG	Y	Y	Hepatotoxicity, peripheral neuropathy	NA	NA
**Danel et al 2015** [13]	Mortality, AIDs defining illness, non-AIDs defining cancer or invasive bacterial disease	Grade 3/4 AE	DAIDS [47]	N	Y	Haematological toxicity, hepatotoxicity, GI intolerance, renal AE, cutaneous reactions, cardiovascular AE, neurological toxicity	NA	NA
**Gupta et al 2019** [14]	Pregnancy related AE	Grade 3-5 AE, mortality, TB incidence	DAIDS [47]	Y	Y	Hepatotoxicity, neuropathy	Pill count	NA

Author	Primary outcome	Secondary outcomes	AE reporting classification	Grade 1-5 AE (Y/N)	Grade >3 SAE	Key adverse events	Adherence reporting method	Treatment completion measurement
**LaCourse et al 2021** [15]	TB infection incidence	Grade 3-5 AE, mortality	DAIDS [47]	N	Y	Hepatotoxicity, neuropathy	NA	NA
**Rifampicin based regimens**								
**Gordin et al 2000** [16]	TB incidence	Hepatotoxicity of any grade, mortality, probable TB	DAIDS [47]	Y	Y	Hepatotoxicity, neuropathy, rash, neutropenia, nausea, vomiting	NA	NA
**Leung et al 2003** [17]	Hepatotoxicity of any grade	NA	NA	N	N	Hepatotoxicity, gout, arthralgia, rash, GI upset, itch	Drug calendar	NA
**Menzies et al 2004** [18]	Percentage of TPT doses taken	AE causing discontinuation, treatment costs	NA	N	N	Hepatotoxicity	Pill count	Completed 80% of doses within 20 weeks (4R), or 43 weeks (9H)
**Tortajada et al 2005** [19]	Safety and tolerability	AE of any grade, adherence, treatment completion	NA	Y	Y	Hepatotoxicity	Pill count	Completed 80% of doses
**Menzies et al 2008** [20]	AE causing discontinuation	Treatment completion	CTCAE [48]	Y	Y	Hepatotoxicity, rash, haematological toxicity, GI intolerance	Pill count	Completed 80% of doses in 120% of time
**Chan et al 2012** [21]	AEs causing discontinuation	Hepatotoxicity of any grade, other causes of treatment cessation, treatment completion	CTCAE [48]	Y	Y	Hepatoxicity	DOT	Not specified
**White et al 2012** [22]	Toxicity, treatment completion	AE of any grade, adherence	NA	Y	Y	Hepatotoxicity, GI intolerance, rash, neurological toxicity, allergy	DOT	Completed 76 doses in 12 months (9H) or 120 doses in 6 months (4R)
**Jimenez-Fuentes et al 2013** [23]	Adherence, AE	TB incidence at 5 years	NA	N	N	Hepatotoxicity, cutaneous reaction, nausea, vomiting, headache, haematological toxicity	Urine metabolites of isoniazid	NA
**Diallo et al 2018** [24]	TB incidence	Grade 3-5 AE, treatment completion	CTCAE [48]	Y	Y	Hepatotoxicity	NA	Completed treatment in 120% of allocated time
**Menzies et al 2018** [25]	TB incidence	Grade 3-5 AE, treatment completion	CTCAE [48]	Y	Y	Hepatotoxicity	NA	Completed treatment in 120% of allocated time

Author	Primary outcome	Secondary outcomes	AE reporting classification	Grade 1-5 AE (Y/N)	Grade >3 SAE	Key adverse events	Adherence reporting method	Treatment completion measurement
**Tamez Torres et al 2023** [26]	AE causing discontinuation	AE of special interest, tolerability, adherence, treatment completion	CTCAE [48]	Y	Y	Hepatotoxicity, GI intolerance, neuropathy, rash, haematological toxicity, seizures, pancreatitis	Pill count	Completed 80% of doses
**Rifapentine based regimens**								
**Sterling et al 2011** [27]	TB incidence	AE causing discontinuation, grade 3-5 AE, mortality, acquired drug resistance, treatment completion	CTCAE [48]	Y	Y	Hepatotoxicity, rash, hypersensitivity	DOT	Not specified
**Villarino et al 2015** [28]	AE causing discontinuation	AE of any grade, mortality, TB incidence at 33 months, treatment completion	NA	Y	Y	Hepatoxicity	DOT	Completed 11/12 doses in 16 weeks (3HP) or 240/270 doses in 52 weeks (9H)
**Denholm et al 2016** [29]	Health system costs	Treatment completion	NA	N	N	NA	NA	Not specified
**Gao et al 2018** [30]	TB incidence	Grade 3-5 AE, treatment completion, mortality, AE causing discontinuation	CTCAE [48]	Y	Y	Hepatotoxicity	DOT	Completed 7-8 doses for (2HP), 11 doses for (3HP)
**Sun et al 2018** [31]	Treatment completion	AE of any grade	DAIDS [47]	Y	Y	Hepatotoxicity, flu-like symptoms, cutaneous reactions, GI intolerance	DOT	Completed treatment within 12 months (9H) or 3 months (3HP)
**Swindells et al 2019** [32]	TB incidence, mortality	Grade 3-5 AE, treatment completion	DAIDS [47]	N	Y	Haematology, hepatotoxicity, GI intolerance, cutaneous reactions, neurological toxicity	NA	Patient reported adherence
**Churchyard et al 2021** [33]	Treatment completion	Grade 3-5 AE, TB incidence, mortality, treatment discontinuation	DAIDS [47]	N	Y	Hypersensitivity, peripheral neuropathy, psychosis, seizures, hepatotoxicity, flu-like symptoms	Pill count	Completed 11 doses of 3HP or at least 167 doses of H
**Ruan et al 2021** [34]	TB incidence	AE of any grade, treatment completion, mortality, acquired drug resistance	CTCAE [48]	Y	Y	Hepatotoxicity, flu-like symptoms	DOT	Completed 11 of 12 doses
**Surey et al 2021** [35]	Treatment completion	AE of any grade	DAIDS [47]	Y	Y	Hepatotoxicity	Pill count	Completed 90% of doses
**Chaisson et al 2023** [36]	CD4 count pre and post treatment	NA	NA	N	N	NA	DOT	NA

Author	Primary outcome	Secondary outcomes	AE reporting classification	Grade 1-5 AE (Y/N)	Grade >3 SAE	Key adverse events	Adherence reporting method	Treatment completion measurement
**Zhang et al 2023** [37]	TB incidence	AE of any grade, treatment completion, AE causing discontinuation, mortality	CTCAE [48]	Y	Y	GI intolerance, neurological toxicity, hypersensitivity, flu-like symptoms	Pill count	Completed 11 of 12 doses
**Chen et al 2024** [38]	Treatment completion	NA	NA	N	N	NA	Pill count	Completed 22 doses of 24 in 16 weeks (HP) or 165 doses of 180 in 8 months (H)
**Huang et al 2024** [39]	Drug reactions	Treatment completion, AESI	NA	Y	Y	Urticaria, flu-like symptoms, hypotension, bronchospasm, hepatotoxicity, conjunctivitis	DOT	Not specified
**Trials investigating multiple regimens**								
**Johnson et al 2001** [40]	TB incidence	Mortality, acquired drug resistance	NA	N	N	NA	NA	NA
**Quigley et al 2001** [41]	TB incidence	Mortality	NA	N	N	NA	NA	NA
**Rivero et al 2003** [42]	TB incidence	AE of any grade, treatment completion, mortality	NA	N	Y	Hepatotoxicity	NA	Completed 80% of doses
**Spyridis et al 2007** [43]	Adherence, treatment completion	AE of any grade, TB incidence	NA	N	N	NA	Urine metabolites of isoniazid	Not specified
**Martinson et al 2011** [44]	TB incidence	AE causing discontinuation, adherence	DAIDS [47]	N	Y	NA	DOT	NA
**Levofloxacin based regimens for MDR/RR-TB**								
**Fox et al 2024** [45]	TB incidence	Grade 3-5 AE, mortality, acquired drug resistance, treatment completion	CTCAE [48]	Y	Y	Hepatotoxicity, prolonged QTc, tendinopathy	NA	Completed 80% of doses within 270 days
**Hesseling et al 2024** [46]	TB incidence	Mortality, grade 3-5 AE	DAIDS [47]	Y	Y	Arthritis, arthralgia, tendinopathy, rash, neurological disturbance	Pill count	NA

ACTG The AIDS clinical trial group, AE adverse events, AESI adverse events of special interest, AIDS acquired immunodeficiency syndrome, CTCAE Common Terminology Criteria for Adverse Events, DAIDS Division of AIDS, DOT directly observed therapy, GI gastrointestinal, H isoniazid, HP isoniazid and rifapentine, N no, NA not applicable, PK pharmacokinetic, QTc corrected QT interval, R rifampicin, SAE severe adverse events, Y yes

Table S3: Primary efficacy outcomes of tuberculosis preventive treatment (TPT) trials (January 2000 - March 2025)

Author (ordered by year of publication)	Regimens (intervention vs control)	Method of excluding TB prior to randomisation	Follow up period (from treatment initiation)	Incident TB assessment	Incident TB in control group n/N (%)	Incident TB in intervention group n/N (%)	Effect size
**Isoniazid-monotherapy**							
**Fitzgerald et al 2000** [1]	12H vs placebo	NA	24 months	Microbiologically[+] or clinically	12/114 (10·5)	3/119 (2·5)	NA
**Agarwal et al 2004** [2]	12H vs no treatment	Clinical assessment, sputum[#]	30 months	Microbiologically or clinically	15/58 (25·8)	3/27 (11·1)	RR 0·36 (95%CI 0·10-1·32)
**Vikrant et al 2005** [3]	12H vs no treatment	NA	Up to 3 years	Microbiologically or clinically	18/55 (32·7)	9/54 (16·7)	RR 0·4 (95% CI 0·17-0·92)
**Naqvi et al 2006** [4]	9H vs no treatment	NA	2 years	Clinically	10/215 (4·6)	1/187 (0·5)	p=0·0001
**Zar et al 2007** [5]	H vs placebo	Clinical assessment, CXR, sputum/lavage microscopy and culture	Median 5·7 months [IQR 2·0-9·7 months]	Microbiologically or clinically	13/131 (9·9)	5/132 (3·8)	HR 0·28 (95% CI 0·10 - 0·78)
**Mohammed et al 2007** [6]	12H vs placebo	Clinical assessment, CXR, sputum microscopy and culture	2 years	Microbiologically or clinically	6/50 (12·0)	9/48 (18·8)	p=0·421
**Madhi et al 2011** [7]	H vs placebo	Clinical assessment, radiology, sputum/lavage microscopy	96-108 weeks	Microbiologically or clinically	38/274 (13·9)	31/273 (11·4)	p=0·40
**Samandari et al 2011** [8]	36H vs 6H + placebo	Clinical assessment	36 months	Microbiologically or clinically	34/989 (3·4)	20/1006 (2·0)	HR 0·57 (95% CI 0·33-0·99)
**Swaminathan et al 2012** [9]	6H vs 36H	Clinical assessment, sputum microscopy and culture	36 months	Microbiologically or clinically	13/339 (3·8)	22/344 (6·4)	aIRR 1·6 (95% CI 0·8-3·2)
**Gray et al 2014** [10]	H vs placebo	Clinical assessment, radiology	34 months	Microbiologically or clinically	7/82 (8·5)	4/85 (4·7)	IRR 0·51 (95% CI 0·15-1·75)
**Kim et al 2015** [11]	9H vs no treatment	Clinical assessment, CXR, sputum microscopy	Mean 21 months (IQR 1·1 - 2·5 years)	Microbiologically or clinically	3/132 (2·2)	0/131 (0·0)	RD 1·22 per 100 person-years (95% CI -0·2-2·65)

Author (ordered by year of publication)	Regimens (intervention vs control)	Method of excluding TB prior to randomisation	Follow up period (from treatment initiation)	Incident TB assessment	Incident TB in control group n/N (%)	Incident TB in intervention group n/N (%)	Effect size
**Rangaka et al 2015** [12]	12H vs placebo	Clinical assessment, sputum culture	Median 2·5 years (up to 3·7 years)	Microbiologically or clinically	58/667 (8·7)	37/662 (5·6)	HR 0·63 (95% CI 0·41-0·94)
**Danel et al 2015** [13]	6H + ART vs ART	NA	30 months	NA	2·5 per 100 person years	1·1 per 100 person years	aHR 0·44 (95% CI 0·28-0·69)
**Gupta et al 2019** [14]	6H during pregnancy vs 6H post-partum	NA	48 weeks	Clinically	3/478 (0·6)	3/477 (0·6)	RD 0·01 (95% CI -0·94 – 0·96)
**LaCourse et al 2021** [15]	12H vs no treatment	NA	12 months	NA	NA*	NA*	NA
**Rifampicin-based regimens**							
**Gordin et al 2000** [16]	2RZ vs 12H	NA	Mean 37 months (range NA)	Microbiologically or clinically	26/792 (3·3)	19/791 (2·4)	RR 0·72 (95%CI 0·40-1·31)
**Leung et al 2003** [17]	2RZ vs 6H	Clinical assessment, CXR, sputum microscopy	Up to ten years	NA	NA*	NA*	NA
**Menzies et al 2004** [18]	4R vs 9H	NA	Treatment duration	NA	NA*	NA*	NA
**Tortajada et al 2005** [19]	2RZ vs 6H	NA	Treatment duration	NA	NA*	NA*	NA
**Menzies et al 2008** [20]	4R vs 9H	NA	Treatment duration	NA	NA*	NA	NA
**Chan et al 2012** [21]	4R vs 6H	Clinical assessment, radiology	Treatment duration	NA	NA*	NA*	NA
**White et al 2012** [22]	4R vs 9H	NA	Treatment duration	NA	NA*	NA*	NA
**Jimenez- Fuentes et al 2013** [23]	3HR vs 6H	Symptoms, CXR, sputum microscopy	5 years	Telephone interview or electronic records	0/294 (0·0)	0/296 (0·0)	NA
**Diallo et al 2018** [24]	4R vs 9H	Clinical assessment, CXR, culture/NAAT if available	16 months	Microbiologically or clinically	2/407 (0·5)	0/422 (0·0)	RD −0·37 cases per 100 person-years; (95% CI, −0·88 to 0·14)
**Menzies et al 2018** [25]	4R vs 9H	Clinical assessment, CXR, culture/NAAT if available	28 months	Microbiologically or clinically	9/3416 (0·3)	8/3443 (0·2)	RD 0·01 cases per 100 person-years (95% CI –0·23 - 0·22)

Author (ordered by year of publication)	Regimens (intervention vs control)	Method of excluding TB prior to randomisation	Follow up period (from treatment initiation)	Incident TB assessment	Incident TB in control group n/N (%)	Incident TB in intervention group n/N (%)	Effect size
**Tamez Torres et al 2023** [26]	3R vs 6H	Clinical assessment, CXR, sputum culture	Treatment duration	NA	NA*	NA	NA
**Rifapentine-based regimens**							
**Sterling et al 2011** [27]	3HP vs 9H	NA	33 months	Microbiologically or clinically	15/3745 (0·4)	7/3986 (0·1)	Absolute difference - 0·24% (upper limit of 95% CI 0·01)
**Villarino et al 2015** [28]	3HP vs 9H	NA	33 months	Microbiologically or clinically	3/434 (0·6)	0/471 (0·0)	RD –0·74% (upper limit of 95% CI 0·32%)
**Denholm et al 2016** [29]	3HP vs 9H	NA	Treatment duration	NA	NA*	NA*	NA
**Gao et al 2018** [30]	3HP, $2H_2P_2$ vs no treatment	CXR	2 years	Microbiologically or clinically	14/1155 (1·21)	10/1284 (0·78) 3HP, 6/1299 (0·46) $2H_2P_2$	HR 0·63 (95% CI 0·27–1·43) (3HP); 0·41 (95% CI 0·15–1·09) (2H2P2)
**Sun et al 2018** [31]	3HP vs 9H	Sputum microscopy and culture	2 years	NA	NA*	NA*	NA
**Swindells et al 2019** [32]	1HP vs 9H	NA	Median 3·3 years (range NA)	Microbiologically or clinically	33/1498 (2·2)	32/1488 (2·2)	Absolute difference -.02 per 100 person-years (upper limit of 95% CI 0·30)
**Churchyard et al 2021** [33]	3HP vs 6H	Clinical assessment, GeneXpert MTB/RIF on sputum	24 months	Microbiologically or clinically	4/357 (1·1)	55/3146 (1·7)	HR 0·96 (95% CI 0·61-1·50)
**Ruan et al 2021** [34]	3HP vs no treatment	Clinical assessment, CXR, sputum culture	37 months	Microbiologically or clinically	19/259 (7·3)	9/254 (3·5)	RR 0·46 (95% CI 0·21-1·05)
**Surey et al 2021** [35]	3HP vs 3RH	NA	Treatment duration	NA	NA*	NA*	NA
**Chaisson et al 2023** [36]	3HP + ART vs ART	Clinical assessment, CRP, GeneXpert Ultra on sputum	2 years	NA	NA*	NA*	NA
**Zhang et al 2023** [37]	6 weeks H_2P_2	Radiology	24 months	Microbiologically or clinically	7/332 (2·1)	8/345 (2·3)	p=0·531

Author (ordered by year of publication)	Regimens (intervention vs control)	Method of excluding TB prior to randomisation	Follow up period (from treatment initiation)	Incident TB assessment	Incident TB in control group n/N (%)	Incident TB in intervention group n/N (%)	Effect size
**Chen et al 2024** [38]	$3H_2P_2$ vs 6H	Clinical assessment, CXR	Treatment duration	NA	NA*	NA*	NA
**Huang et al 2024** [39]	1HP vs 3HP	NA	Treatment duration	NA	NA*	NA*	NA
**Trials investigating multiple regimens**							
**Johnson et al 2001** [40]	3HR, 3HRZ, 6H vs placebo	Clinical assessment, sputum microscopy/culture	2 years	Microbiologically or clinically	64/787 (8·1)	51/931 (5·5) 6H 22/556 (4·0) 3HR 15/462 (3·2) 3HRZ	aRR 0·46 (95% CI 0·29-0·71)
**Quigley et al 2001** [41]	6H, 3RZ vs placebo	Clinical assessment, CXR	Mean 3 years (up to several years)	Microbiologically or clinically	40/940 (4·3)	29/957 (3·0) 6H 33/903 (3·7) 3RZ	NA
**Rivero et al 2003** [42]	6H, 3RH, 2RZ vs no treatment	NA	2 years	Microbiologically or clinically	4/77 (5·2)	3/83 (3·6) 6H 3/82 (3·7) 3HR 1/77 (1·3) 2RZ	RR 1·07 (95% CI 0·24-4·8) (6H), RR 0·98 (95% CI 0·22-4·4) (3HR), RR 0·39 (95% CI 0·04-3·48) (2RZ)
**Spyridis et al 2007** [43]	3HR, 4HR vs 9H	NA	3 years	Microbiologically or clinically	0/232 (0·0)	0/238 (0·0) 3HR 0/236 (0·0) 4HR	NA
**Martinson et al 2011** [44]	3HP, 3HR, 6 years H vs 6H	Clinical assessment, CXR, sputum culture	Mean 4 years (up to 6 years)	Microbiologically or clinically	22/327 (6·7)	24/328 (7·3) 3HP 24/329 (7·3) RH 8/164 (4·9) 6yH	p>0·05
**Levofloxacin-based regimens in MDR/RR-TB**							
**Fox et al 2024** [45]	6Lfx vs placebo	Clinical assessment, radiology, sputum NAAT	30 months	Microbiologically or clinically	11/1018 (1·1)	6/1023 (0·6)	IRR 0·55 (95% CI 0·19-1·62)
**Hesseling et al 2024** [46]	6Lfx vs placebo	Clinical assessment, CXR, sputum culture	72 weeks	Microbiologically or clinically	12/469 (2·6)	5/453 (1·1)	HR 0·44 (95% CI 0·15-1·25)

ART antiretroviral therapy, CI confidence interval, CRP c-reactive peptide, CXR chest x-ray, HP isoniazid and rifapentine weekly, H_2P_2 isoniazid and rifapentine twice weekly, HR hazard ratio, IGRA interferon gamma release assay, IRR incidence ratio rate, IQR interquartile range, Lfx levofloxacin, NA not available, NAAT nucleic acid amplification test, R rifampicin, RD rate difference, RR risk ratio, TB tuberculosis, Z pyrazinamide.

* TB incidence not available as an outcome

#sputum testing method not specified

[+] Methods for microbiological diagnosis of tuberculosis varied widely across studies and included smear, GeneXpert and culture

References

1. Fitzgerald DW, Desvarieux M, Severe P, Joseph P, Johnson WD, Jr., Pape JW. Effect of post-treatment isoniazid on prevention of recurrent tuberculosis in HIV-1-infected individuals: a randomised trial. *Lancet* 2000; **356**(9240): 1470-4.
2. Agarwal SK, Gupta S, Dash SC, Bhowmik D, Tiwari SC. Prospective randomised trial of isoniazid prophylaxis in renal transplant recipient. *Int Urol Nephrol* 2004; **36**(3): 425-31.
3. Vikrant S, Agarwal SK, Gupta S, et al. Prospective randomized control trial of isoniazid chemoprophylaxis during renal replacement therapy. *Transplant infectious disease* 2005; **7**(3-4): 99-108.
4. Naqvi R, Akhtar S, Noor H, et al. Efficacy of isoniazid prophylaxis in renal allograft recipients. *Transplantation proceedings* 2006; **38**(7): 2057-8.
5. Zar HJ, Cotton MF, Strauss S, et al. Effect of isoniazid prophylaxis on mortality and incidence of tuberculosis in children with HIV: randomised controlled trial. *Bmj* 2007; **334**(7585): 136.
6. Mohammed A, Myer L, Ehrlich R, Wood R, Cilliers F, Maartens G. Randomised controlled trial of isoniazid preventive therapy in South African adults with advanced HIV disease. *International journal of tuberculosis and lung disease* 2007; **11**(10): 1114-20.
7. Madhi SA, Nachman S, Violari A, et al. Primary isoniazid prophylaxis against tuberculosis in HIV-exposed children. *New England Journal of Medicine* 2011; **365(1)**: 21-31.
8. Samandari T, Agizew TB, Nyirenda S, et al. 6-month versus 36-month isoniazid preventive treatment for tuberculosis in adults with HIV infection in Botswana: a randomised, double-blind, placebo-controlled trial. *Lancet* 2011; **377**(9777): 1588-98.
9. Swaminathan S, Menon PA, Gopalan N, et al. Efficacy of a six-month versus a 36-month regimen for prevention of tuberculosis in HIV-infected persons in India: a randomized clinical trial. *PLoS One* 2012; **7**(12): e47400.
10. Gray DM, Workman LJ, Lombard CJ, et al. Isoniazid preventive therapy in HIV-infected children on antiretroviral therapy: a pilot study. *International journal of tuberculosis and lung disease* 2014; **18**(3): 322-7.
11. Kim SH, Lee SO, Park IA, et al. Isoniazid treatment to prevent TB in kidney and pancreas transplant recipients based on an interferon-γ-releasing assay: an exploratory randomized controlled trial. *J Antimicrob Chemother* 2015; **70**(5): 1567-72.
12. Rangaka MX, Wilkinson RJ, Boulle A, et al. Isoniazid plus antiretroviral therapy to prevent tuberculosis: a randomised double-blind, placebo-controlled trial. *Lancet* 2014; **384**(9944): 682-90.
13. Danel C, Moh R, Gabillard D, et al. A Trial of Early Antiretrovirals and Isoniazid Preventive Therapy in Africa. *N Engl J Med* 2015; **373**(9): 808-22.
14. Gupta A, Montepiedra G, Aaron L, et al. Isoniazid Preventive Therapy in HIV-Infected Pregnant and Postpartum Women. *New England journal of medicine* 2019; **381**(14): 1333-46.
15. LaCourse SM, Richardson BA, Kinuthia J, et al. A Randomized Controlled Trial of Isoniazid to Prevent Mycobacterium tuberculosis Infection in Kenyan Human Immunodeficiency Virus-Exposed Uninfected Infants. *Clinical infectious diseases* 2021; **73**(2): e337-e44.
16. Gordin F, Chaisson RE, Matts JP, et al. Rifampin and pyrazinamide vs isoniazid for prevention of tuberculosis in HIV-infected persons: an international randomized trial. Terry Beirn Community Programs for Clinical Research on AIDS, the Adult AIDS Clinical Trials Group, the Pan American Health Organization, and the Centers for Disease Control and Prevention Study Group. *Jama* 2000; **283**(11): 1445-50.

17. Leung CC, Law WS, Chang KC, et al. Initial Experience on Rifampin and Pyrazinamide vs Isoniazid in the Treatment of Latent Tuberculosis Infection among Patients with Silicosis in Hong Kong. *Chest* 2003; **124(6)**: 2112-8.

18. Menzies D, Dion MJ, Rabinovitch B, Mannix S, Brassard P, Schwartzman K. Treatment completion and costs of a randomized trial of rifampin for 4 months versus isoniazid for 9 months. *American Journal of Respiratory and Critical Care Medicine* 2004; **170(4)**: 445-9.

19. Tortajada C, Martinez-Lacasa J, Sanchez F, et al. Is the combination of pyrazinamide plus rifampicin safe for treating latent tuberculosis infection in persons not infected by the human immunodeficiency virus? *International Journal of Tuberculosis and Lung Disease* 2005; **9(3)**: 276-81.

20. Menzies D, Long R, Trajman A, et al. Adverse events with 4 months of rifampin therapy or 9 months of isoniazid therapy for latent tuberculosis infection: A randomized trial. *Annals of Internal Medicine* 2008; **149(10)**: 689-97.

21. Chan PC, Yang CH, Chang LY, et al. Latent tuberculosis infection treatment for prison inmates: a randomised controlled trial. *International journal of tuberculosis and lung disease* 2012; **16**(5): 633-8.

22. White MC, Tulsky JP, Lee JR, et al. Isoniazid vs. rifampin for latent tuberculosis infection in jail inmates: toxicity and adherence. *J Correct Health Care* 2012; **18**(2): 131-42.

23. Jimenez-Fuentes MA, De Souza-Galvao ML, Mila Auge C, Solsona Peiro J, Altet-Gomez MN. Rifampicin plus isoniazid for the prevention of tuberculosis in an immigrant population. *International Journal of Tuberculosis and Lung Disease* 2013; **17(3)**: 326-32.

24. Diallo T, Adjobimey M, Ruslami R, et al. Safety and Side Effects of Rifampin versus Isoniazid in Children. *N Engl J Med* 2018; **379**(5): 454-63.

25. Menzies D, Adjobimey M, Ruslami R, et al. Four Months of Rifampin or Nine Months of Isoniazid for Latent Tuberculosis in Adults. *New England journal of medicine* 2018; **379**(5): 440-53.

26. Tamez-Torres KM, Mongua-Rodríguez N, Ferreyra-Reyes L, et al. Safety and Tolerability of Six Months of Isoniazid Plus Pyridoxine or Three Months of Rifampicin for Tuberculosis among Subjects with Diabetes Mellitus: A Randomized Trial. *Microorganisms* 2023; **11**(8).

27. Sterling TR, Villarino ME, Borisov AS, et al. Three months of rifapentine and isoniazid for latent tuberculosis infection. *N Engl J Med* 2011; **365**(23): 2155-66.

28. Villarino ME, Scott NA, Weis SE, et al. Treatment for preventing tuberculosis in children and adolescents: a randomized clinical trial of a 3-month, 12-dose regimen of a combination of rifapentine and isoniazid. *JAMA pediatrics* 2015; **169**(3): 247-55.

29. Denholm JT, McBryde ES, Eisen D, et al. SIRCLE: a randomised controlled cost comparison of self-administered short-course isoniazid and rifapentine for cost-effective latent tuberculosis eradication. *Intern Med J* 2017; **47**(12): 1433-6.

30. Gao L, Zhang H, Xin H, et al. Short-course regimens of rifapentine plus isoniazid to treat latent tuberculosis infection in older Chinese patients: a randomised controlled study. *The european respiratory journal* 2018; **52**(6).

31. Sun HY, Huang YW, Huang WC, et al. Twelve-dose weekly rifapentine plus isoniazid for latent tuberculosis infection: A multicentre randomised controlled trial in Taiwan. *Tuberculosis* 2018; **111**: 121-6.

32. Swindells S, Ramchandani R, Gupta A, et al. One Month of Rifapentine plus Isoniazid to Prevent HIV-Related Tuberculosis. *N Engl J Med* 2019; **380**(11): 1001-11.

33. Churchyard G, Cárdenas V, Chihota V, et al. Annual Tuberculosis Preventive Therapy for Persons With HIV Infection : a Randomized Trial. *Annals of internal medicine* 2021; **174**(10): 1367-76.

34. Ruan QL, Huang XT, Yang QL, et al. Efficacy and safety of weekly rifapentine and isoniazid for tuberculosis prevention in Chinese silicosis patients: a randomized controlled trial. *Clinical microbiology and infection* 2021; **27**(4): 576-82.

35. Surey J, Stagg HR, Yates TA, et al. An open label, randomised controlled trial of rifapentine versus rifampicin based short course regimens for the treatment of latent tuberculosis in England: the HALT LTBI pilot study. *BMC infectious diseases* 2021; **21**(1): 90.

36. Chaisson LH, Semitala FC, Nangobi F, et al. Viral suppression among adults with HIV receiving routine dolutegravir-based antiretroviral therapy and 3 months weekly isoniazid-rifapentine. *Aids* 2023; **37**(7): 1097-101.
37. Zhang H, Xin H, Du Y, et al. Tuberculosis preventive treatment among individuals with inactive tuberculosis suggested by untreated radiographic abnormalities: a community-based randomized controlled trial. *Emerg Microbes Infect* 2023; **12**(1): e2169195.
38. Chen H, Zhang H, Cheng J, et al. Adherence to preventive treatment for latent tuberculosis infection in close contacts of pulmonary tuberculosis patients: A cluster-randomized controlled trial in China. *International Journal of Infectious Diseases* 2024; **147(no pagination)**.
39. Huang HL, Lee MR, Lee CH, et al. One-month daily and three-month weekly rifapentine plus isoniazid are comparable in completion rate and safety for latent tuberculosis infection in non-HIV Population: a randomized controlled trial. *Clinical Microbiology and Infection* 2024; **30(11)**: 1410-7.
40. Johnson JL, Okwera A, Hom DL, et al. Duration of efficacy of treatment of latent tuberculosis infection in HIV-infected adults. *Aids* 2001; **15**(16): 2137-47.
41. Quigley MA, Mwinga A, Hosp M, et al. Long-term effect of preventive therapy for tuberculosis in a cohort of HIV-infected Zambian adults. *Aids* 2001; **15**(2): 215-22.
42. Rivero A, López-Cortés L, Castillo R, et al. [Randomized clinical trial investigating three chemoprophylaxis regimens for latent tuberculosis infection in HIV-infected patients]. *Enferm Infecc Microbiol Clin* 2007; **25**(5): 305-10.
43. Spyridis NP, Spyridis PG, Gelesme A, et al. The effectiveness of a 9-month regimen of isoniazid alone versus 3- and 4-month regimens of isoniazid plus rifampin for treatment of latent tuberculosis infection in children: Results of an 11-year randomized study. *Clinical Infectious Diseases* 2007; **45(6)**: 715-22.
44. Martinson NA, Barnes GL, Moulton LH, et al. New regimens to prevent tuberculosis in adults with HIV infection. *New England Journal of Medicine* 2011; **365(1)**: 11-20.
45. Fox GJ, Nhung NV, Cam Binh N, et al. Levofloxacin for the Prevention of Multidrug-Resistant Tuberculosis in Vietnam. *N Engl J Med* 2024; **391**(24): 2304-14.
46. Hesseling AC, Purchase SE, Martinson NA, et al. Levofloxacin Preventive Treatment in Children Exposed to MDR Tuberculosis. *N Engl J Med* 2024; **391**(24): 2315-26.
47. National Institute of Health. DAIDS Adverse Experience Reporting System (DAERS). 2025. https://rsc.niaid.nih.gov/sites/default/files/DAERS-User-Instructional-Guide.pdf.
48. National Cancer Institute Division of cancer treatment and diagnosis. Common Terminology Criteria for Adverse Events (CTCAE). 2025. https://ctep.cancer.gov/protocoldevelopment/electronic_applications/ctc.htm.
